# Case Report: Extragonadal Yolk Sac Tumors Originating From the Endometrium and the Broad Ligament: A Case Series and Literature Review

**DOI:** 10.3389/fonc.2021.672434

**Published:** 2021-06-11

**Authors:** Xianzhong Cheng, Qian Zhao, Xia Xu, Wenwen Guo, Hongyuan Gu, Rui Zhou, Chen Chen, Dawei Ma, Yinan Wu, Jing Ni, Xiaoxiang Chen

**Affiliations:** ^1^ Department of Gynecologic Oncology, Jiangsu Cancer Hospital, Jiangsu Institute of Cancer Research, The Affiliated Cancer Hospital of Nanjing Medical University, Nanjing, China; ^2^ Department of Chemotherapy, Jiangsu Cancer Hospital, Jiangsu Institute of Cancer Research, The Affiliated Cancer Hospital of Nanjing Medical University, Nanjing, China; ^3^ Department of Pathology, The Second Affiliated Hospital of Nanjing Medical University, Nanjing, China; ^4^ Department of Pathology, Jiangsu Cancer Hospital, Jiangsu Institute of Cancer Research, The Affiliated Cancer Hospital of Nanjing Medical University, Nanjing, China

**Keywords:** extragonadal, yolk sac tumor, endometrium, broad ligament, bleomycin, etoposide and platinum

## Abstract

Yolk sac tumors (YSTs) of the endometrium and the broad ligament are very rare, with only 29 cases and one case of each other reported before in the English literature. Due to lack of standard guidelines, the treatment strategies of these diseases are controversial. Here, we share two cases of YSTs originating from the endometrium and the broad ligament respectively and review related literature. A 35-year-old woman was diagnosed with endometrial YST in our center and underwent surgery followed by chemotherapy with BEP (bleomycin, cisplatin and etoposide) regimen for six courses. After follow-up for 21 months, there is still no evidence of relapse. Another 36-year-old woman was admitted to our department with YST of the broad ligament. She was treated with surgery followed by chemotherapy with BEP regimen and was lost to follow-up after completing therapy. The case of endometrial YST we shared was similar to cases reported before, while the case with YST of the broad ligament we shared was the second case reported worldwide. Both of these two cases were treated with surgery combined with chemotherapy with BEP regimen.

## Introduction

Yolk sac tumor (YST), also known as endodermal sinus tumor, is a malignant germ cell tumor usually accompanied by elevated serum alpha-fetoprotein (AFP). Most YSTs occurred in the gonads and the midline of the body of young women, young children, and infants (1). Approximately 10% YSTs arise in extra-gonadal sites for female patients, including sacrococcygeal region, retroperitoneum, mediastinum, pineal gland, stomach, liver, omentum, pelvis, and genital system (2). YSTs originating from the endometrium and the broad ligament are very rare and difficult to distinguish those from endometrial cancer and other pelvic mass preoperatively. Up to now, there have been only 16 case reports and two case series of primary endometrial YST reported before in English literature (1, 3–19). In addition, there was only one case of primary YST of the broad ligament reported in 1970 (20). Here, we share two cases of primary YSTs, including endometrial YST and YST of the broad ligament respectively and review related articles.

This study was approved by the ethics committee of The Affiliated Cancer Hospital of Nanjing Medical University. Informed consent was obtained from patients for the publication of any potentially identifying information and images included in this article.

## Case Presentations

### Case 1

**Figure 1:**
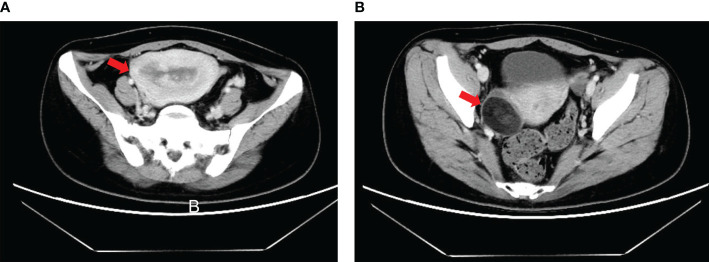
Computed tomography (CT) scan of patient with endometrial YST preoperatively. **(A)** Enlarged uterus equal to pregnancy for 2 months accompanied by occupancy in the uterus cavity; **(B)** A 3-cm mass of uneven density in the right adnexa.

A 35-year-old woman was admitted to a local hospital for sudden abnormal uterine bleeding and chronic extended menstrual period in March 2019. Diagnostic curettage was performed at the local hospital, the result of which was endometrial carcinoma not excluding clear cell carcinoma. After consultation with the pathological department of our center, endometrial carcinoma was considered for this patient. Then she was hospitalized in the gynecologic oncology department of our center. Pelvic examination revealed enlarged uterus equal to pregnancy for 2 months without other obvious abnormalities. Computed tomography (CT) scan indicated that the image was similar to that of endometrial cancer with the myometrium invasion, accompanied by a 3-cm mass of uneven density in the right adnexa **(** **Figure 1** **)**. The serum AFP was 9,152 ng/ml, while other serum tumor markers including carbohydrate antigen (CA)72-4, CA12-5, CA19-9, CA15-3, neuron specific enolase (NSE), carcinoembryonic antigen (CEA), and *β*-human chorionic gonadotropin (*β*-HCG) were 14.39, 29.39, 13.48, and 9.86 U/ml, 16.48 and 0.540 ng/ml and 0.477 mIU/ml, respectively. Based on results of these examinations, the patient was diagnosed with endometrial cancer and scheduled for surgery immediately.

**Figure 2:**
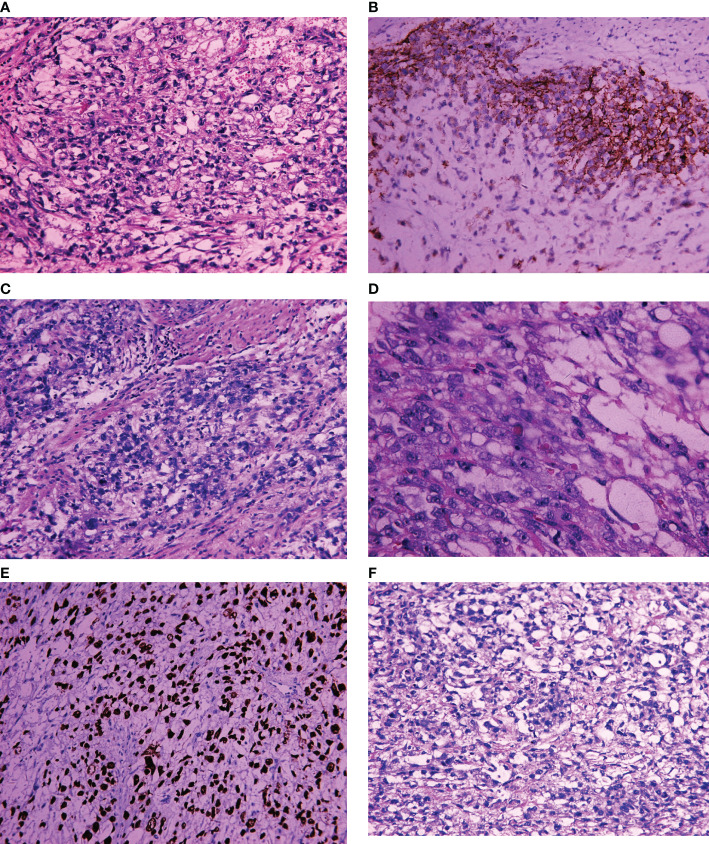
Light microscopic appearance and immunohistochemical staining of endometrial YST. **(A)** Yolk sac structure (H and E, 10 × 40); **(B)** CD117+; **(C)** Germ cell tumor (GCT) cytology (H and E, 10 × 40); **(D)** Hyaloid drop (H and E, 10 × 40); **(E)** SALL4+; **(F)** Schiller–Duval body positive (H and E, 10 × 40).

On April 2, 2019, exploratory laparotomy was performed including total hysterectomy, bilateral adnexectomy, omental resection, pelvic lymphadenectomy and paraaortic lymphadenectomy. Intraoperatively, the enlarged uterus remained at the size of 2 months after conception contaminant with an approximately 3-cm mass in the right adnexa. Postoperative pathology of gross appearance showed grayish-white mass at the endometrium and a teratoma in the right adnexa, which were about 6 cm × 5 cm × 1 cm and 3 × 2 cm × 2 cm in size, respectively. Microscopically, yolk sac tumor of the endometrium with both less than half of the full muscularis layer invasion and intravascular invasion was revealed. No metastasis was found at any other sites, including bilateral adnexa, omentum, cervix, and lymph nodes. Mature teratoma was confirmed for the mass at the right adnexa without any other abnormalities. Immunohistochemical staining of endometrial YST yielded the following positive results: SALL4+, AFP+, AE1/AE3+, CD117+, Ki-67 positivity rate of approximately 80% and patchy positivity for VIM. Immunohistochemistry yielded negative results for cytokeratin (CK)7, OCT (octamer-binding transcription factor)3/4, CD30, CD34, estrogen receptor, and progesterone receptor. In addition, endodermal sinusoidal (Schiller–Duval body) and hyaloid drop were also observed microscopically **(** **Figure 2** **)**. According to the FIGO (International Federation of Gynecology and Obstetrics) staging classification published in 2018, the patient belongs to stage IA (21).

Two weeks after operation, serum AFP was 231.7 ng/ml. From April 2019 to August 2019, four courses of chemotherapy with BEP (bleomycin, cisplatin and etoposide) regimen were administered for this patient. During the period of chemotherapy, the serum AFP decreased obviously, and all serum tumor markers had fallen to normal after completing chemotherapy for two cycles. Adverse events of chemotherapy were not serious which included nausea, vomiting, and other chemotherapy related reactions. Follow-up after surgery and chemotherapy including pelvic examination, serum tumor markers, CT scanning, and pelvic ultrasound was also conducted regularly, and the patient was still alive and free of disease for 21 months after surgery.

### Case 2

A 36-year-old woman was hospitalized at a local hospital for pelvic mass found by physical examination in November 2017. Exploratory laparotomy was performed at that hospital immediately. Intraoperatively, a free mass at approximately 6 cm × 5 cm × 5 cm in size was observed at the bottom of the right pelvis and adjacent to the uterosacral ligament without any other abnormalities. Due to the examination of frozen sections showing germ cell tumor and consultation with relatives of this patient, pelvic mass resection, biopsy of bilateral ovaries and partial omentum resection were performed. Postoperative pathology reported yolk sac tumor of the pelvic mass, normal biopsy from ovaries and normal tissue of omentum. From December 2017 to June 2018, six courses of chemotherapy with BEP regimen were administrated for her. After five cycles of chemotherapy, serum tumor markers of the patient were all normal, but her alanine aminotransferase (ALT) was elevated nearly to 600 IU/L. She was treated with hepatoprotective therapy for 20 days before completing the sixth cycle chemotherapy. Unfortunately, the serum AFP increased again after sixth cycle of chemotherapy.

**Figure 3:**
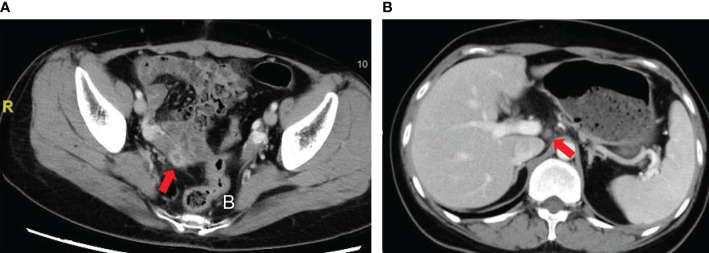
Computed tomography (CT) scan of patient with YST originating from the broad ligament. **(A)** A 2-cm mass at the right pelvis; **(B)** Slightly enlarged lymph nodes.

Due to increase of serum AFP, the patient was admitted to our center in July 2018. Positron emission tomography–computed tomography (PET/CT) demonstrated a round-like mass at the right pelvis accompanied by increased [18F]-fluorodeoxyglucose (FDG) metabolism. CT scan also indicated a 2-cm mass at the right pelvis **(** **Figure 3** **)**. Considering relapse of the disease, the patient was hospitalized at our department for a second surgery. During the operation, a dumbbell-shaped, hard, and smooth mass at approximately 3 × 2 × 1.5 cm in size was found at the posterior lobe of right broad ligament and near the uterus. No abnormalities were observed at any other sites of the pelvic and abdominal cavity. After mass resection and right adnexectomy, frozen section was examined and demonstrated yolk sac tumor of broad ligament and normal right adnexa. In consideration of normal adnexa and consultation with her relatives, no other resection was conducted.

**Figure 4:**
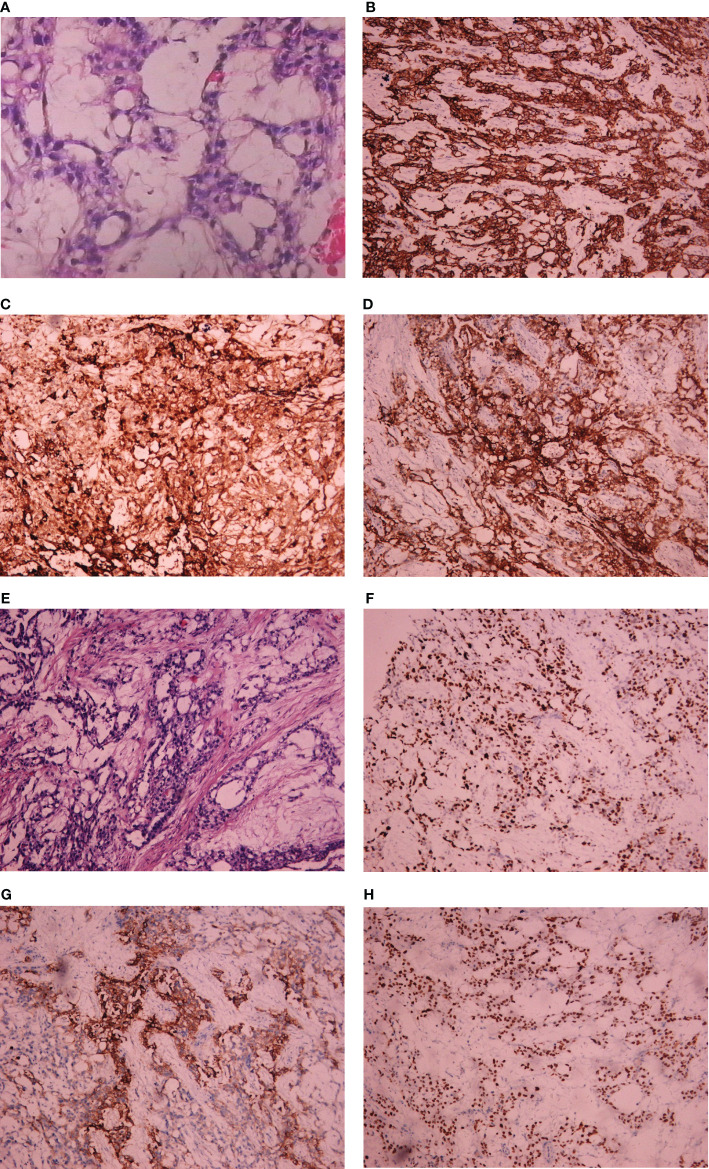
Light microscopic appearance and immunohistochemical staining of YST originating from the broad ligament. **(A)** Yolk sac tumor (H and E, 10 × 40); **(B)** AE1/AE3+; **(C)** AFP++; **(D)** GPC-3(++); **(E)** Yolk sac tumor (H and E, 10 × 40); **(F)** Ki-67 positivity rate of approximately 70%; **(G)** PLAP negative; **(H)** SALL4 +++.

Postoperatively, pathology report confirmed yolk sac tumor of broad ligament again and normal adnexa. Immunohistochemical staining yielded the following positive results: SALL4 +++, AFP ++, GPC-3 ++, AE1/AE3 +, Ki-67 positivity rate of approximately 70%, patchy positivity for CD117 and VIM+/−. Immunohistochemistry yielded negative results for CK7, CD30, PAX-8, and PLAP **(** **Figure 4** **)**. Considering diagnosis with yolk sac tumor, four courses of chemotherapy with BEP regimen were administrated for this patient from August 2018 to November 2018. After completing three cycles of chemotherapy, the serum AFP was 15.39 ng/ml. During the period of chemotherapy, adverse events were not serious which included decreased white blood cells and neutrophils. CT and magnetic resonance image (MRI) scan showed thickening of the area of the right adnexa not excluding relapse. She was discharged after four courses of chemotherapy and the follow-up failed later.

## Literature Review and Discussions

Herein, we share a case series of primary YSTs originating from the endometrium and the broad ligament. In order to summarize all reported cases and compare with our cases, we search the related literature using the following combination of MeSH-terms and keywords: “extragonadal”, “yolk sac tumor”, “YST”, “primary”, “endodermal sinus tumor”, “endometrium”, “endometrial”, “broad ligament” in Pubmed, Scopus, and Cochrane Library. Furthermore, references of selected articles were also retrieved to obtain all potentially relevant literature. After review of the English literature, there were only 29 cases of primary endometrial YST including 16 case reports and two case series published before. In addition, extragonadal YST of the broad ligament was reported only once before, which originated from the left broad ligament, so the case we shared was the first case of YST from the right broad ligament in the world (20). All reported cases were summarized in **Table S1**.

Previous literature reported that YSTs usually occurred in the young population. Among 29 cases of primary endometrial YST, the median age was 59 years (range 24–87 years) which seemed to be in contradiction with the theory of early occurrence of YSTs before. There were 11 cases of endometrial YST from M.D. Anderson Cancer Center, an institution that primarily serves an adult patient population, which possibly contributes to an older median age (15). The median age of these 11 cases and the other 18 reported cases were 64 years (range 55–87 years) and 40 years (range 24–68 years). The patient we shared was 35 years old, which was similar to reported cases except the series from M.D. Anderson Cancer Center. Similarly, the age of primary YST arising from the broad ligament reported before and our case was 18 years and 36 years, respectively (20).

The diagnosis of primary YSTs of the endometrium and the broad ligament was still a challenge. On one hand, the literature reported before indicated that symptoms of primary endometrial YST included abnormal vaginal bleeding [82.8% (24/29)], abdominal pain [13.8% (4/29)], abdominal distension [3.4% (1/29)], and abnormal vaginal discharge [3.4% (1/29)]. The patient we reported was admitted to our center due to abnormal vaginal bleeding which was the most common symptom of primary endometrial YST. On the other hand, patients with primary YST usually had an increased level of serum AFP. Among 17 patients with available serum AFP, 88.2% (15/17) of them had an increased value, but only 11.8% (2/17) of them had a normal value. The cases we showed also had an elevated serum AFP, which was a significant symbol for diagnosis of primary YSTs and discrimination from other diseases such as endometrial cancer and undetermined pelvic mass.

Due to lack of standard guidelines, the treatment strategies of primary YSTs from the endometrium and the broad ligament are controversial. According to reported cases of primary endometrial YST, surgery combined with chemotherapy was the main strategy for reported cases of primary endometrial YST. Among 26 patients with available surgical information, all of them underwent surgery, and 16 of them were administrated with chemotherapy, yielded a percentage of 100% (26/26) and 94.1% (16/17), except for nine without available information of chemotherapy. For those with detailed surgical information, the proportion of total abdominal hysterectomy, modified hysterectomy, and modified radical hysterectomy was 88.2% (15/17), 5.9% (1/17), and 5.9% (1/17), respectively. There were only three cases that suffered ovarian metastasis, all of whom were diagnosed with stage FIGO IV disease (4, 5, 12). This may indicate that patients with young age and early stage endometrial YST can preserve their ovaries if they have fertility requirements. The percentages of bilateral salpingo-oophorectomy, single salpingo-oophorectomy, bilateral ovary biopsies plus fallopian tube resection and preservation of bilateral adnexa were 76.5% (13/17), 5.9% (1/17), 5.9% (1/17), 5.9% (1/17), and 5.9% (1/17). In terms of lymphadenectomy, 58.8% (10/17) of cases underwent pelvic lymph node dissection, and 29.4% (5/17) of them also underwent paraaortic lymphadenectomy. The postoperative FIGO stage ranged from IA to IVB including 35.7% (10/28) for stage I, 17.9% (5/28) for stage II, 21.4% (6/28) for stage III, and 25.0% (7/28) for stage IV. Pathological results also demonstrated that Schiller–Duval (SD) bodies were observed for 62.5% (15/24) of patients with available information, but 16.7% (4/24) of them are rare. Interestingly, the median ages of patients with positive SD bodies, rare SD bodies, and no SD bodies were 44 years (range 24–68 years), 59.5 years (range 42–68 years), and 64 years (range 59–87 years) separately. This demonstrated that SD bodies may decrease as patients being older.

Among 16 patients with available information of chemotherapy, the percentages of BEP regimen, VAC regimen, and other regimens were 56.3% (9/16), 12.5% (2/16), and 31.3% (5/16) individually. In terms of chemotherapy regimen, BEP regimen was the most common regimen. The two cases we shared were also treated with the BEP regimen, but their prognosis was markedly different. The case with endometrial YST was free of disease for 21 months, while the disease from the broad ligament relapsed soon after primary therapy, which indicates that BEP regimen may be not an excellent choice for patients with YST of the broad ligament. Furthermore, radiotherapy was also conducted for 19.2% (5/26) of those who underwent surgery, including 15.4% (4/26) with chemotherapy and 3.8% (1/26) without chemotherapy.

Without fertility desire, the case of primary endometrial YST we shared was treated with surgery, including total hysterectomy, bilateral adnexectomy, omental resection, pelvic lymphadenectomy, and paraaortic lymphadenectomy, followed by chemotherapy with BEP regimen, which was the most common strategy for endometrial YST reported before. After follow-up for 21 months, no evidence of disease progression was indicated till now. The outcome of treatment and follow-up differed from each other among all cases published before. As for YST from the broad ligament, the case published before underwent laparotomy, had widespread metastases in the pelvic, and refused to accept chemotherapy and radiotherapy later (20). The case of YST from the broad ligament we shared relapsed soon after primary surgery combined with chemotherapy and underwent secondary surgery/chemotherapy treatment strategy. Therefore, both of the two cases of YST originating from the broad ligament had a poor prognosis. The reason why YST from the broad ligament had worse prognosis than endometrial YST may be derived from the mechanisms of its occurrence. According to reported cases, four hypotheses for YST originating from the endometrium are as follows: 1) metastasis from other sites; 2) residual tissues from incomplete abortion; 3) abnormal differentiation of somatic cells; 4) abnormal migration of primordial germ cells (1, 9). However, the mechanisms of YST from the broad ligament need to be explored. As for female patients with extragonadal YSTs, there was still lack of standard guidelines. According to NCCN guidelines for ovarian YSTs, the most common regimen is BEP regimen, while some patients with recurrent disease can also be treated with high-dose chemotherapy and stem cell transplantation as a salvage therapy (22). Considering both the ovarian YST and the broad ligament YST occurring outside the uterus and metastasizing easily, the treatment strategies suited for ovarian disease may be also suited for the broad ligament YST and need to be explored in the future.

## Conclusions

YSTs originating from the endometrium and the broad ligament are very rare, especially YST from the broad ligament. There are several symptoms of endometrial YST, the most common of which is abnormal vaginal bleeding, usually accompanied by elevated serum AFP. The case of YST from the broad ligament we shared was the second case in the English literature. Due to lack of standard guidelines, the treatment strategies are controversial and needed to be explored.

## Data Availability Statement

The original contributions presented in the study are included in the article/**Supplementary Material**. Further inquiries can be directed to the corresponding author.

## Ethics Statement

The studies involving human participants were reviewed and approved by The Affiliated Cancer Hospital of Nanjing Medical University. Written informed consent was obtained from the individual for the publication of any potentially identifiable images or data included in this article.

## Author Contributions

XXC and JN were involved in the identification and selection of patient cases. XZC was involved in drafting the manuscript. XZC and QZ were involved in collecting patients’ information, conducting follow-up. XXC, WWG, and JN reviewed and edited the manuscript. QZ, XX, and HYG were involved in the management of patients and reviewed the manuscript. RZ and CC carried out literature review. DWM and YNW carried out the diagnosis of pathological results and reviewed the manuscript. All authors contributed to the article and approved the submitted version.

## Funding

This study was supported by grants from the National Natural Science Foundation of China (No. 81472441, 81501205), Institute level project of Jiangsu Cancer Hospital (No. ZM201804), Beijing Kanghua Foundation for the Development of Traditional Chinese and Western Medicine -Le Fund (KH-2020-LJJ-021), and Jiangsu Province Maternal and Child Health Research Project (F202004).

## Conflict of Interest

The authors declare that the research was conducted in the absence of any commercial or financial relationships that could be construed as a potential conflict of interest.

## References

[1] Song L Wei X Wang D Yang K Qie M Yin R. Primary Yolk Sac Tumor Originating From the Endometrium: A Case Report and Literature Review. Medicine (2019) 98(15):e15144. 10.1097/MD.0000000000015144 30985686 PMC6485813

[2] Pasternack T Shaco-Levy R Wiznitzer A Piura B Research G. Extraovarian Pelvic Yolk Sac Tumor: Case Report and Review of Published Work. J Obstet Gynaecol Res (2008) 34(4 Pt 2):739–44. 10.1111/j.1447-0756.2008.00725.x 18840194

[3] Abhilasha N Bafna U Pallavi V Rathod P Krishnappa S. Primary Yolk Sac Tumor of the Endometrium: A Rare Entity. Indian J Cancer (2014) 51(4):446. 10.4103/0019-509X.175315 26842154

[4] Clement PB Young RH Scully REJC. Extraovarian Pelvic Yolk Sac Tumors. Cancer (1988) 62(3):620–6. 10.1002/1097-0142(19880801)62:3<620::aid-cncr2820620330>3.0.co;2-p 3292037

[5] Damato S Haldar K McCluggage WG. Primary Endometrial Yolk Sac Tumor With Endodermal-Intestinal Differentiation Masquerading as Metastatic Colorectal Adenocarcinoma. Int J Gynecol Pathol (2016) 35(4):316–20. 10.1097/PGP.0000000000000236 26598980

[6] Ji M Lu Y Guo L Feng F Wan X Xiang YJO. Endometrial Carcinoma With Yolk Sac Tumor-Like Differentiation and Elevated Serum β-hCG: A Case Report and Literature Review. OncoTargets Ther (2013) 6:1515. 10.2147/OTT.S51983 PMC3810345 24187502

[7] Joseph MG Fellows FG Hearn SAJC. Primary Endodermal Sinus Tumor of the Endometrium. A Clinicopathologic, Immunocytochemical, and Ultrastructural Study. Cancer (1990) 65(2):297–302. 10.1002/1097-0142(19900115)65:2<297::aid-cncr2820650219>3.0.co;2-e 1688508

[8] Lin S-W Hsieh S-W Huang S-H Liang H-S Huang C-Y. Gynecology. Yolk Sac Tumor of Endometrium: A Case Report and Literature Review. Taiwan J Obstet Gynecol (2019) 58(6):846–8. 10.1016/j.tjog.2019.09.020 31759539

[9] Lu T Qi L Ma Y Lu G Zhang X Liu P. Primary Yolk Sac Tumor of the Endometrium: A Case Report and Review of the Literatures. Arch Gynecol Obstet (2019) 300(5):1177–87. 10.1007/s00404-019-05309-3 31549219

[10] Oguri H Sumitomo R Maeda N Fukaya T Moriki T. Primary Yolk Sac Tumor Concomitant With Carcinosarcoma Originating From the Endometrium: Case Report. Gynecol Oncol (2006) 103(1):368–71. 10.1016/j.ygyno.2006.04.024 16814851

[11] Ohta M Sakakibara K Mizuno K Kano T Matsuzawa K Tomoda Y. Successful Treatment of Primary Endodermal Sinus Tumor of the Endometrium. Gynecol Oncol (1988) 31(2):357–64. 10.1016/s0090-8258(88)80015-5 3169623

[12] Ozler A Dogan S Mamedbeyli G Rahatli S Haberal AN Dursun P. Primary Yolk Sac Tumor of Endometrium: Report of Two Cases and Review of Literature. J Exp Ther Oncol (2015) 11(1):5–9. 26259383

[13] Patsner B. Primary Endodermal Sinus Tumor of the Endometrium Presenting as “Recurrent” Endometrial Adenocarcinoma. Gynecol Oncol (2001) 80(1):93–5. 10.1006/gyno.2000.6011 11136577

[14] Pileri S Martinelli G Serra L Bazzocchi FJO. Gynecology. Endodermal Sinus Tumor Arising in the Endometrium. Obstet Gynecol (1980) 56(3):391–6. 6158720

[15] Ravishankar S Malpica A Ramalingam P Euscher ED. Yolk Sac Tumor in Extragonadal Pelvic Sites. Am J Surg Pathol (2017) 41(1):1–11. 10.1097/PAS.0000000000000722 27631522

[16] Rossi R Stacchiotti D Bernardini MG Calvieri G Voi RL. Primary Yolk Sac Tumor of the Endometrium: A Case Report and Review of the Literature. Am J Obstet Gynecol (2011) 204(4):e3–4. 10.1016/j.ajog.2010.12.014 21345404

[17] Spatz A Bouron D Pautier P Castaigne D Duvillard P. Primary Yolk Sac Tumor of the Endometrium: A Case Report and Review of the Literature. Gynecol Oncol (1998) 70(2):285–8. 10.1006/gyno.1998.5036 9740707

[18] Shokeir MO Noel SM Clement PB. Malignant Müllerian Mixed Tumor of the Uterus With a Prominent Alpha-Fetoprotein-Producing Component of Yolk Sac Tumor. Mod Pathol (1996) 9(6):647–51.19. JMpaojotUS, Canadian Academy of Pathology I. 8782202

[19] Wang C Li G Xi L Gu M Ma D. Obstetrics. Primary Yolk Sac Tumor of the Endometrium. Int J Gynaecol Obstet (2011) 114(3):291–3. 10.1016/j.ijgo.2011.03.020 21696729

[20] Huntington RW Jr. Bullock WKJC. Yolk Sac Tumors of Extragonadal Origin. Cancer (1970) 25(6):1368–76. 10.1002/1097-0142(197006)25:6<1368::aid-cncr2820250615>3.0.co;2-p 5422911

[21] Amant F Mirza MR Koskas M Creutzberg CL. Cancer of the Corpus Uteri. Int J Gynaecol Obstet (2018) 143(Suppl 2):37–50. 10.1002/ijgo.12612 30306580

[22] Reddy Ammakkanavar N Matei D Abonour R Einhorn LH. High-Dose Chemotherapy for Recurrent Ovarian Germ Cell Tumors. J Clin Oncol (2015) 33(2):226–7. 10.1200/JCO.2014.59.4325 25452440

