# Supplementary material for: Case Report: Extragonadal Yolk Sac Tumors Originating From the Endometrium and the Broad Ligament: A Case Series and Literature Review
Source: Front Oncol. 2021 Jun 11;11:672434. doi: 10.3389/fonc.2021.672434 (PMC8240588; doi:10.3389/fonc.2021.672434)
Supplement: Supplementary file 1 [file DataSheet_1.docx]

Supplementary Material

# Supplementary Table 1 Summary of clinicopathologic features of primary YSTs originating from the endometrium and the broad ligament.

| Case  (Ref) | Age  (yrs) | Primary site | Symptoms | AFP level | Surgery | FIGO stage | SD bodies | ovarian metastasis | Chemotherapy | Radiotherapy | Follow-up time(mo) | Follow-up results |
| --- | --- | --- | --- | --- | --- | --- | --- | --- | --- | --- | --- | --- |
| 1 (4) | 24 | Endometrium | Abd pain | 3600ng/mL | CISH+BSO | IVA | Yes | Yes | VAC 6CY | Yes | 24 | DOD |
| 2 (11) | 27 | Endometrium | AVB | 1580ng/mL | TAH+BSO+OMT | IA | Yes | No | VAC | No | 14 | NED |
| 3 (9) | 27 | Endometrium | AVB | 800ng/mL | TAH+ Bilateral fallopian tube resection+ BOB+OMB+PLND+PALND | IA | NA | No | DC 6CY | No | 14 | NED |
| 4 (14) | 28 | Endometrium | AVB, Abd pain | 380ng/mL | TAH+BSO | IB | Yes | No | VCR, VLB, CTX, ADR, MDX, 5-Fu, MPA | No | 2;8 | REC; DOD |
| 5 (6) | 28 | Endometrium | AVB | 1522ng/mL | TAH+BSO+OMT+PLND+appendectomy+partial resection of the sigmoid colon with anastomosis | IV | NA | No | PTX, ADM, CDDP, CBDCA, MTX, Act-D, VP-16, BLM, pingyangmycin, VCR, FUDR, oxaliplatin, CPA | No | 10 | AWD |
| 6 (19) | 29 | Endometrium | AVB | 3593.4ng/mL | Modified hysterectomy+left adnexa+PLND+PALND | II | Yes | No | BEP 4CY | No | 39 | DOD |
| 7 (16) | 30 | Endometrium | AVB | 1762ng/mL | TAH | II | Yes | OP | BEP 3CY | No | 72 | NED |
| 8 (3) | 31 | Endometrium | AVB | 222ng/mL | TAH+BSO+OMT+PLND+PALND | IA | NA | No | BEP 4CY | No | 24 | NED |
| 9 (1) | 38 | Endometrium | AVB | Normal | TAH+BSO+LD+OMT+appendectomy | IVB | NA | No | BEP 6CY | No | 24 | NED |
| 10 (7) | 42 | Endometrium | AVB | 18530ng/mL | TAH+BSO | IA | Rare | No | PVB 4CY | No | 24 | NED |
| 11 (12) | 44 | Endometrium | AVB | 27522ng/mL | TH+BSO+OMT+PLND+PALND | IB | Yes | NA | BEP 3CY | No | 6 | NED |
| 12 (17) | 49 | Endometrium | AVB | Normal | TAH+BSO+PLND | IA | Yes | No | No | Yes | 28 | NED |
| 13 (15) | 55 | Endometrium | AVB | NA | Yes | Ⅱ | Rare | NA | NA | Yes | 16 | DOD |
| 14 (12) | 57 | Endometrium | Abd pain | 29214ng/mL | TAH+BSO+OMT+PLND+PALND | IVB | Yes | Yes | BEP 2CY | No | 2 | DOD |
| 15 (13) | 59 | Endometrium | Abd pain | 25385ng/mL | TAH+BSO+OMT+PLND | NA | Yes | NA | BEP 4CY+EP 2CY | No | 16 | REC; AWD |
| 16 (15) | 59 | Endometrium | AVB | NA | NA | ⅠB | No | NA | NA | NA | NA | LTF |
| 17 (15) | 61 | Endometrium | AVB | NA | Yes | ⅠA | No | NA | NA | No | 8 | AWD |
| 18 (15) | 62 | Endometrium | AVB | NA | Yes | ⅠB | No | NA | NA | No | 30 | AWD |
| 19 (5) | 63 | Endometrium | AVB | NA | TAH + LSO + OMT +appendicectomy | IVB | NA | Yes | BEP 3CY | No | 6 | NED |
| 20 (15) | 63 | Endometrium | AVB | NA | Yes | ⅢC | No | NA | NA | Yes | 5 | NED |
| 21 (18) | 64 | Endometrium | Abd distension | 15918ng/mL | NA | IVB | Rare | NA | NA | No | 2.5 | DOD |
| 22 (15) | 64 | Endometrium | AVB | NA | Yes | ⅢA | No | NA | NA | Yes | 23 | DOD |
| 23 (10) | 65 | Endometrium | AVD | 2306ng/mL | Modified RH+BSO+PLND | IIIC | Yes | No | TP 5CY | No | NA | NA |
| 24 (15) | 68 | Endometrium | AVB | NA | Yes | Ⅳ | Rare | NA | NA | No | 14 | DOD |
| 25 (8) | 68 | Endometrium | AVB | 133.4 ng/mL | TAH+OMT+BSO+PLND+PALND | Ⅱ | Yes | No | BEP 6CY | No | >6 | NED |
| 26 (15) | 71 | Endometrium | AVB | NA | Yes | ⅢA | No | NA | NA | No | 19 | DOD |
| 27 (15) | 77 | Endometrium | AVB | NA | NA | ⅢC | No | NA | NA | NA | NA | LTF |
| 28 (15) | 77 | Endometrium | AVB | NA | Yes | ⅢC | No | NA | NA | No | 17 | AWD |
| 29 (15) | 87 | Endometrium | AVB | NA | Yes | Ⅱ | No | NA | NA | No | 7 | AWD |
| 30 (20) | 18 | Left broad ligament | Abd pain | NA | Yes | NA | NA | NA | No | No | 4 | DOD |

Ref, References; Abd, abdominal; AVB, abnormal vaginal bleeding; AVD, abnormal vaginal discharge; CISH, classical intrafascial supracervical hysterectomy; BSO, bilateral salpingo-oophorectomy; TAH, total abdominal hysterectomy; RH, radical hysterectomy; OMT, omentectomy; OMB, omentum multipoint biopsy; BOB, bilateral ovary biopsies; PLND, pelvic lymph node dissection; PALND, para-aortic lymph node dissection; Ret, microcystic/reticular; Pap, papillary; So, solid; Tub, tubular; Gld, glandular; Hep, hepatoid; SD body, Schiller–Duval bodies; OP, ovarian preservation; CY, cycles; AWD, alive with disease; DOD, died of disease; LTF, lost to follow-up; NED, no evidence of disease; NA, not available.

## Supplementary Figure 1 Treatment timelines of YSTs originating from the endometrium **(A)** and the broad ligament **(B)**.

**(A)**


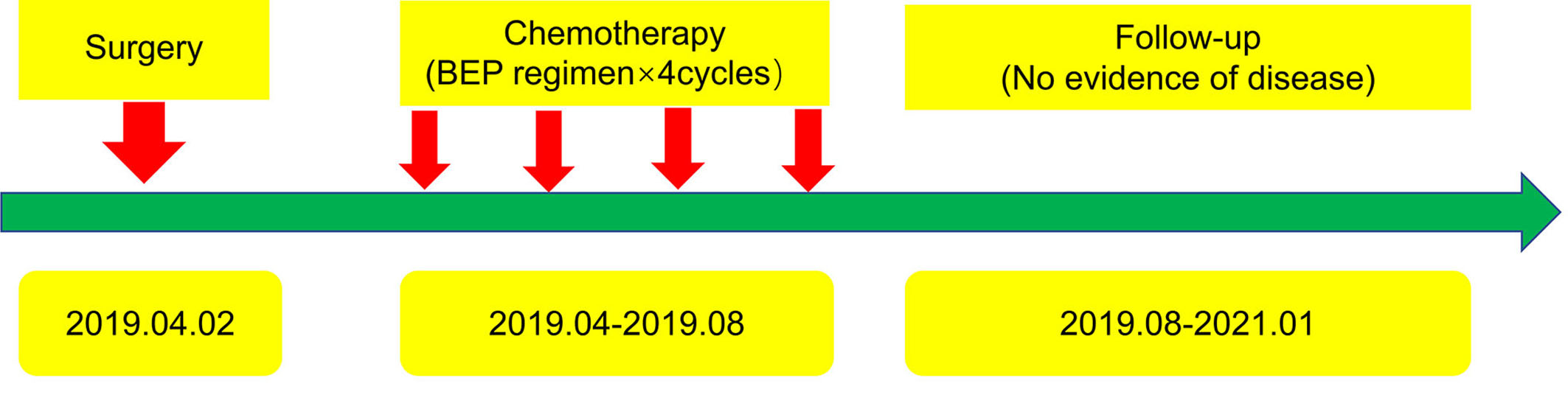


**(B)**

**
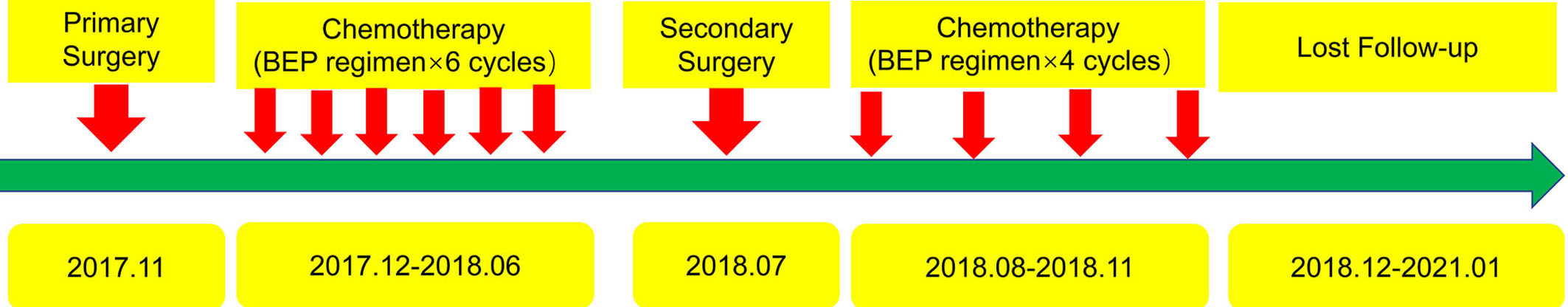
**
